# Predictors of outcomes in hematopoietic cell transplantation for Fanconi anemia

**DOI:** 10.1038/s41409-023-02121-1

**Published:** 2023-10-17

**Authors:** Maria Cancio, Alexandre G. Troullioud Lucas, Marc Bierings, Elizabeth Klein, Moniek A. de Witte, Frans J. Smiers, Dorine Bresters, Jaap Jan Boelens, Stephanie E. Smetsers

**Affiliations:** 1 Transplantation and Cellular Therapy, MSK Kids, Department of Pediatrics, Memorial Sloan Kettering Cancer Center, New York, NY USA; 2 Princess Máxima Center for Pediatric Oncology, Utrecht, the Netherlands; 3 Department of Pediatric Oncology/Hematology, Wilhelmina Children’s Hospital/University Medical Center Utrecht, Utrecht, the Netherlands; 4 Department of Hematology, University Medical Center Utrecht, Utrecht, the Netherlands; 5 Department of Pediatric Hematology and Stem Cell Transplantation, Willem Alexander Children’s Hospital/Leiden University Medical Center, Leiden, the Netherlands

**Keywords:** Anaemia, Myelodysplastic syndrome, Risk factors, Acute myeloid leukaemia, Stem-cell therapies

## Abstract

Allogeneic hematopoietic cell transplantation (HCT) remains the only cure for the hematologic manifestations of Fanconi anemia (FA). We performed retrospective predictor analyses for HCT outcomes in FA for pediatric and young adult patients transplanted between 2007 and 2020 across three large referral institutions. Eighty-nine patients, 70 with bone marrow failure +/− cytogenetic abnormalities, 19 with MDS/AML, were included. Five-year overall survival (OS) was 83.2% and event-free survival (EFS) was 74%. Age ≥19, HLA mismatch and year of HCT were multivariable predictors (MVPs) for OS, EFS and treatment-related mortality (TRM). In the pediatric group, TCD was a borderline MVP (*P* = 0.059) with 5-year OS of 73.0% in TCD vs. 100% for T-replete HCT. The cumulative incidence of day 100 grade II-IV aGvHD and 5-year cGvHD were 5.6% and 4.6%, respectively. Relapse in the MDS/AML subgroup occurred in 4 patients (16%). Graft failure was seen in 9 patients (TCD 6/37 [16%]; T-replete 3/52 [5.7%]). Six patients developed malignancy after HCT. Survival chances after HCT for FA are excellent and associated with high engrafted survival and low toxicity. Age ≥19, HLA mismatch, year of transplant and ‘TCD in the <19 years group’ (although borderline) were found to be negative predictors for survival.

## Introduction

Fanconi anemia (FA) is a rare, inherited bone marrow failure (BMF) syndrome, characterized by congenital abnormalities, pancytopenia and predisposition to malignancies, especially myelodysplastic syndrome (MDS), acute myeloid leukemia (AML) and squamous cell carcinoma (SCC) [1]. While gene therapy trials for FA are ongoing, allogeneic hematopoietic cell transplantation (allo-HCT) is still the only standard of care curative option for hematologic malignancies of FA, but does not prevent the occurrence of solid tumors, mainly head and neck SCC. It has also been argued that graft versus host disease (GvHD) after allo-HCT might contribute to the development of solid tumors in these patients [2].

Outcomes after allo-HCT have improved significantly over the last 30 years by optimizing preparative regimens to decrease toxicities related to the high sensitivity of FA patients to DNA alkylating agents, specifically by the introduction of fludarabine [3]. Furthermore, graft-versus-host-disease (GvHD) prophylaxis, human leucocyte antigen (HLA)-typing and supportive care have contributed to the improved outcomes [4, 5].

Recent series describing outcomes after allo-HCT for FA have included relatively limited numbers of patients [6, 7], or, when larger numbers of patients were included, have been registry studies, including data from centers with low volume of allo-HCT for FA [5].

In this study we describe our retrospective predictor analysis for outcomes of allo-HCT in FA patients performed between 2007 and 2020 across three large FA referral institutions using different transplant platforms.

## Methods

Data of patients with FA and BMF or FA related myeloid malignancies undergoing their first HCT at Memorial Sloan Kettering Cancer Center (New York, USA), the University Medical Center Utrecht/Princess Máxima Center for Pediatric Oncology (Utrecht, the Netherlands) and Leiden University Medical Center (Leiden, the Netherlands) between 2007 and 2020, with follow up through December 31, 2020, was collected prospectively. We conducted retrospective analysis of this data, with no restrictions in terms of age, gender, HLA matching, conditioning regimen, cell source (cord blood [CB], bone marrow [BM] and peripheral blood stem cells [PBSCs]) and/or graft source or manipulation. As part of clearance to proceed to transplant patients were required to have a performance score above 70 (Karnofsky or Lansky depending on age). Stem cell donors were defined as matched donors (HLA match 10/10, 8/8 [BM and PBSC] or 6/6 [CB]), including matched related donors (MRD) and matched unrelated donors (MUD), or mismatched donors (HLA match ≤9/10, or ≤5/6 for CB). HLA typing was performed by licensed laboratories according to state-of-the-art technologies. Supportive care was similar among the centers, including monitoring for viruses, and did not change over time. The study was approved by the Institutional Review Boards of all three institutions. Patients signed a general transplant consent as well as a data collection consent. For these specific analyses the need for an additional informed consent was waived.

Indication for HCT was defined as moderate or severe BMF (with or without cytogenetic changes), MDS or AML. For patients with MDS/AML, the strategy from all centers was to proceed to HCT without prior treatment of MDS/AML. The severity of the cytopenia was defined per Camitta et al. 1976 classification [8]. MDS was defined as the presence of cytogenetic abnormalities and dysplastic changes in greater than 10% of cells morphologically [9], based on reports from the institutional licensed pathologists. This definition was used to differentiate MDS from BMF with cytogenetic changes. For the definition of MDS/AML relapse, we also used reports from the institutional licensed pathologists.

### Outcomes

Main outcomes of interest were overall survival (OS) and event free survival (EFS). OS time was defined as time from allo-HCT to time of death from any cause or time of last follow-up for survivors. EFS time was defined as time from allo-HCT to time of event or time of last follow-up for patient who did not have an event. Events were defined as relapse (MDS/AML), graft failure (GF) and treatment-related mortality (TRM). GF was defined by either no engraftment at day 30 (for PBSC and BM) or 42 (for CB) post-transplant or loss of the graft after initial engraftment (secondary graft failure) [10]. We used the definition of engraftment of the first of three consecutive days with an absolute neutrophil count greater than 0.5 × 10^9^/L.

Other outcomes of interest were TRM (death not due to relapse), acute graft versus host disease (aGvHD) at day 100 as defined by CIBMTR criteria, extensive chronic GvHD (cGvHD) defined by NIH criteria, engraftment, and post-transplant malignancies.

### Statistical analysis

Continuous variables are displayed as median and range, discrete variables as counts and proportions. Cox proportional hazard models were used to study possible impact of variables on the outcomes of interest. Variables considered were age, gender, indication (BMF with or without cytogenetic abnormalities versus MDS/AML), HLA matching, conditioning regimen, graft source, graft manipulation, FANC complementation group and year of transplant before versus after the median (2014). The results are presented as hazard ratios (HRs), 95% confidence intervals (95% CIs), and log-likelihood test *P* values. Factors were assessed in univariable models first and subsequently entered into multivariable (MV) models if *P* ≤ 0.05. The Kaplan-Meier method was used to visualize and analyze the main outcomes of interest OS and EFS. For analysis of cumulative incidences of TRM, aGvHD and cGvHD Fine and Gray models for competing risk were used.

## Results

### Patient and transplant characteristics

A total of 89 consecutive FA patients were included. Patient and allo-HCT characteristics are summarized in Table 1 and Supplementary Tables S1 and S2. Subsets of patients have been reported previously; 29 patients by Smetsers et al. [3], and 10 patients by Mehta et al. [6]. Median age at transplant for the entire cohort was 9.2 (range 1.7–44) years. Sixteen of 89 patients were adults (≥19 years), who were transplanted at a median age of 30.2 (range 22.8–44) years. For the 73 (82%) patients <19 years old, median age at transplant was 8 (range 1.7–18.9) years. In total, 15 patients had MDS, and 4 patients had AML at time of transplant, the rest of the patients had BMF with or without cytogenetic changes. Of the patients <19 years old, 12 (16.4%) had MDS/AML while in the group ≥19 years old this number was 7 (43.8%).

**Table 1:** Patient and HCT characteristics.

Total *n* = 89	*n* (%)		*n* (%)
Center		Conditioning regimen	
Leiden University Medical Center	12 (13.5)	Cy/Flu	51 (57.3)
Memorial Sloan Kettering Cancer Center	35 (39.3)	Bu^d^/Cy/Flu	26 (29.2)
University Medical Center Utrecht/Princess Maxima Center for Pediatric Oncology	42 (47.2)	TBI/Cy/Flu	11 (12.4)
Age at HCT		Cy/Thiotepa	1 (1.1)
Median (years)	9.2	Serotherapy	
Range (years)	1.7-44	ATG	84 (94.4)
Gender		Alemtuzumab	1 (1.1)
Female	33 (37.1)	None	4 (4.5)
Male	56 (62.9)	HLA matching	
Complementation group		Matched	59 (66.3)
*FANCA*	52 (58.4)	Mismatched	30 (33.7)
*FANCC*	22 (24.7)	Donor	
*FANCE*	2 (2.2)	MUD	37 (41.6)
*FANCG*	3 (3.4)	MRD	22 (24.7)
Other^a^	4 (4.5)	MMUD	20 (22.5)
Unknown	6 (6.7)	MMRD	10 (11.2)
Disease status at time of HSCT		Stem cell source	
Bone marrow failure +/− cytogenetic changes^b^	70 (78.7)	Bone marrow	45 (50.6)
MDS/AML^c^	19 (21.3)	Peripheral blood	32 (36)
Follow-up		Cord blood	12 (13.5)
Median (years)	3.6	Graft manipulation	
Range (years)	0.9–14.3	Unmanipulated/conventional^e^	52 (58.4)
		Ex-vivo T cell depletion^f^	37 (41.6)

^a^In the group ‘Other’ we included one of each of the following: *FANCB*, *FANCD2*, *FANCL*, and *FANCM*.

^b^Cytogenetic changes observed in patients with bone marrow failure were: 3q26/EVI1 rearrangement, +X, 7-, 7q-, and 20q-.

^c^Cytogenetic changes observed in patients with MDS/AML were: 3q26/EVI1 rearrangement, 1q+, 1q24+, 1q25+, 3q27+, 6p25-, 7q-, 9-, 9+, 11q-, 12p-, 12p13-, 15q+.

^d^BU target was 18–22 mg*h/L at MSKCC and 30 mg*h/L at LUMC and UMCU/PMC.

^e^GvHD prophylaxis used was dependent on graft source/manipulation. For bone marrow we used cyclosporine (CsA) and methotrexate (*n* = 31), CsA and mycophenolate mofetil (MMF; *n* = 3), CsA/MMF/prednisone (*n* = 1), CsA/MMF/tacrolimus/prednisone (*n* = 1), CsA/methotrexate/tacolimus (*n* = 1), MMF/prednisone (*n* = 1), MMF alone (*n* = 1), or CsA alone (*n* = 1). For cord blood we used CsA/prednisone (*n* = 9) or CsA alone (*n* = 3).

^f^T-cell depletion devices used were Isolex (*n* = 8) and CliniMACS (*n* = 29).

The most commonly used conditioning regimen was cyclophosphamide/fludarabine (Cy/Flu). The addition of busulfan (Bu) to the conditioning regimen was dependent on local institutional practice; at Memorial Sloan Kettering Cancer Center the Bu target was 18–22 mg*hr/L, while in the European centers it was 30 mg*hr/L. A minority of patients received total body irradiation (TBI; 12.4%), in a range of 3–4.5 Gy. Almost all patients received serotherapy (95.5%), and more than half of the patients had a matched donor. Bone marrow was the stem cell source in about half of the transplants, followed by peripheral blood and cord blood respectively. Ex vivo T-cell depletion (TCD) was used in 37 (41.6%) of the transplants, of which 20 (54.0%) were HLA-mismatched. Of the T-replete allo-HCTs, 40 (77%) were from bone marrow and 12 (23% [10 mismatched; 19.2%]) from cord blood.

### Outcomes

**Fig. 1:**
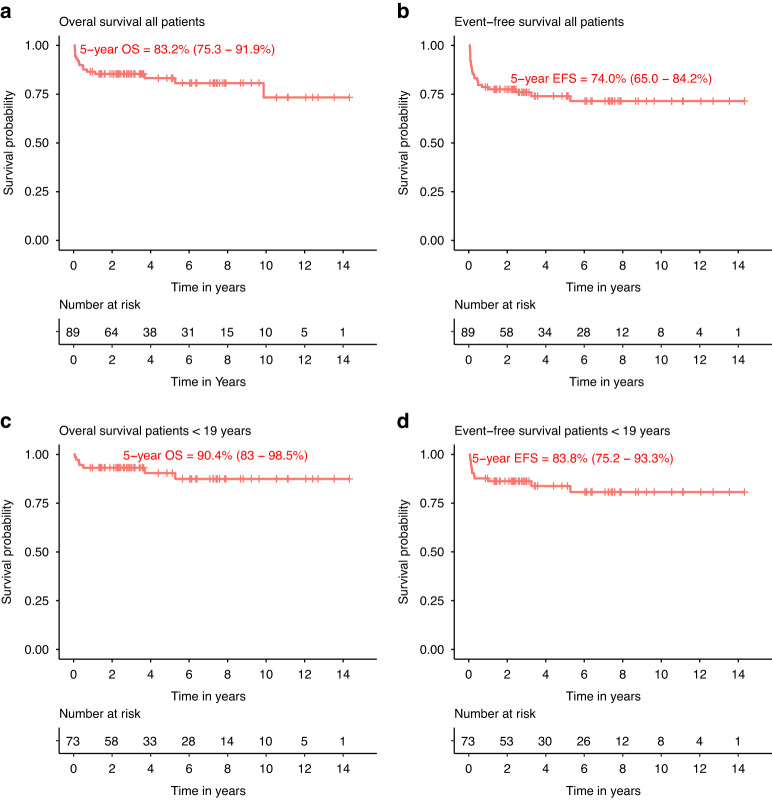
Outcomes for full cohort. Overall survival (**a**) and event-free survival (**b**) in our full cohort and overall survival (**c**) and event-free survival (**d**) in patients younger than 19 years of age at time of transplant.

For the full cohort, 5-year OS and EFS were 83.2% (75.3–91.9%) and 74% (65–84.2%), respectively (Fig. 1a, b). Sixteen of 89 patients died; causes of death were infection (fungal [*n* = 2] and viral [*n* = 5]), GvHD (*n* = 3), multiorgan failure (*n* = 2), secondary malignancy (SCC of the tongue and urothelial carcinoma; *n* = 2), graft failure (*n* = 1) and progression of leukemia (*n* = 1). 5-year OS by donor type was 91.9% (83.5–100%) for MUD, 87% (70.8–100%) for MRD, 74.7% (57.7–96.6%) for mismatched unrelated donor (MMUD) and 55.6% (31–99.7%) for mismatched related donor (MMRD). Patients with BMF +/− cytogenetic changes had a significantly better 5-year OS compared to patients with MDS/AML (90% [83.2–97.3%] versus 58.6% [38.2–90.1%], *p* = 0.015).

**Fig. 2:**
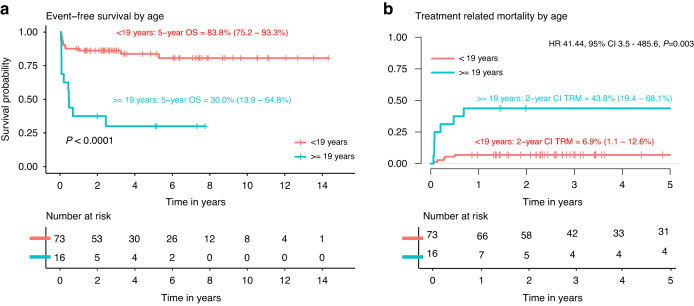
Outcomes by age. Event-free survival (**a**) and treatment related mortality (death by other cause than relapse; **b**) by age <19 years compared to age ≥19 years.

In univariable Cox proportional hazard analysis for OS, EFS and TRM; age, indication for transplant, graft manipulation, conditioning regimen, HLA match and year of transplant were found to be significant predictors. Table 2 summarizes the multivariable analysis of the full cohort for these outcomes. For OS age ≥19 (HR 13.4, 95% CI 2.3–77, *P* = 0.004), HLA mismatch (HR 4.7, 95% CI 1.3–16.6, *P* = 0.02) and transplant in 2014 or later (HR 0.12, 95% CI 0.02–0.57, *P* = 0.008) were found to be multivariable predictors. Likewise, for EFS the same multivariable predictors were found; age ≥19 (HR 8.8, 95% CI 2.6–30.2, *P* < 0.001, Fig. 2a), HLA mismatch (HR 6.2, 95% CI 2.3–16.7, *P* < 0.001) and HCT in 2014 of later (HR 0.31, 95% CI 0.11–0.84, *P* = 0.02). Finally, these covariates were also found to be multivariable predictors of TRM; age ≥19 (HR 29.9, 95% CI 2.8–319, *P* = 0.005, Fig. 2b), HLA mismatch (HR 10, 95% CI 1.8–54.2, *P* = 0.008) and transplant in 2014 or later (HR 0.06, 95% CI 0.01–0.52, *P* = 0.011).

**Table 2:** Multivariable Cox PH analysis all patients (*n* = 89).

	Overall survival		Event-free survival		Treatment-related mortality	
	HR (95% CI)	*P*	HR (95% CI)	*P*	HR (95% CI)	*P*
Age at transplant						
≤8 years	1		1		1	
8–18.9 years	3.1 (0.54–17.6)	0.20	2.1 (0.6–7.3)	0.25	7.5 (0.67–83.4)	0.10
≥19 years	13.4 (2.3–77)	0.004*	8.8 (2.6–30.2)	<0.001*	29.9 (2.8–319)	0.005*
Transplant indication						
BMF	1		1		1	
MDS/AML	0.51 (0.12–2.1)	0.35	0.36 (0.12–1.1)	0.08	0.21 (0.04–1.2)	0.072
Ex vivo T-cell depletion						
No	1		1		1	
Yes	1.4 (0.26–7.4)	0.69	2.5 (0.57–10.8)	0.22	1.6 (0.21–11.8)	0.66
Conditioning regimen^a^						
Including busulfan	1		1		1	
Without busulfan	0.33 (0.05–2.2)	0.25	0.68 (0.15–3.1)	0.62	0.17 (0.01–2.03)	0.16
Including TBI	0.87 (0.24–3.2)	0.84	0.91 (0.28–3.0)	0.88	0.68 (0.14–3.3)	0.64
HLA match						
Matched	1		1		1	
Mismatched	4.7 (1.3–16.6)	0.02*	6.2 (2.3–16.7)	<0.001*	10 (1.8–54.2)	0.008*
HCT year						
<2014	1		1		1	
≥2014	0.12 (0.02–0.57)	0.008*	0.31 (0.11–0.84)	0.02*	0.06 (0.01–0.52)	0.011*

^a^Although busulfan target AUCs were different between MSKCC and the centers in the Netherlands, only 3 patients in the Netherlands received busulfan as part of their conditioning regimen, so no further sub-analyses on busulfan exposure could be performed.

*P < 0.05.

In total, 84 patients (94.4%) had sustainable neutrophil recovery. Median time to neutrophil recovery was 15 (range: 7–35) days. Five patients (5.6%) had primary graft failure (graft source: CB [*n* = 2], T-cell depleted PBSC [*n* = 3]; conditioning regimen: Cy/Flu [*n* = 2], Bu/Cy/Flu [*n* = 2], TBI/Cy/Flu [*n* = 1]); 4 patients (4.5%) had secondary graft failure (graft source: BM [*n* = 1], T-cell depleted PBSC [*n* = 3]; conditioning regimen: Cy/Flu [*n* = 2], Bu/Cy/Flu [*n* = 2]).

The cumulative incidence of day 100 grade II-IV aGvHD, grade III-IV aGvHD, and 5-year extensive cGvHD were 5.6%, 2.2% and 4.6%, respectively. Relapse in the MDS/AML subgroup was only seen in 4 patients (16%). GF was seen in 9 patients (TCD 6/37 [16%]; T-replete 3/52 [5.7%]). Six patients developed malignancy after allo-HCT at a median time of 6.3 (range 0.9–13) years after transplant; 4 had received T-replete, 2 ex vivo TCD transplants. The malignancies were SCC of the oral mucosa (*n* = 3), basal cell carcinoma (*n* = 1), urothelial (*n* = 1) and hepatocellular carcinoma (*n* = 1). Preceding these diagnoses, 2 patients had grade I, 1 had grade II, and 1 had grade III aGvHD and limited cGvHD.

**Fig. 3:**
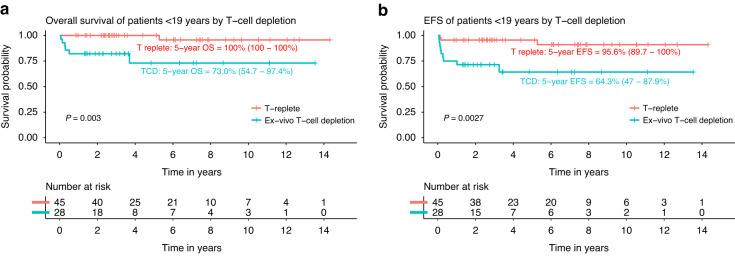
Outcomes in patients <19 years old by T-cell depletion. Overall survival (**a**) and event-free survival (**b**) in patients younger than 19 years, by T-replete transplants versus ex vivo T-cell depleted transplants.

In the pediatric patients (age <19 years, *n* = 73), 5-year OS and EFS were 90.4% (83–98.5%) and 83.8% (75.2–93.3%), respectively (Fig. 1c, d). Graft manipulation (TCD) and HLA-match were found to be univariable predictors for the main outcomes of interest: OS and EFS. In multivariable analysis including these variables (Table 3), TCD was found to be the only borderline multivariable predictor for inferior OS suggesting an 8-fold increased risk of an event (HR 8.4, 95% CI 0.9–76.6, *P* = 0.059) with 5-year OS of 73.0% (54.7–97.4%) in TCD vs 100% for T-replete HCT (Fig. 3a). HLA mismatch was a predictor of worse EFS (HR 9.8, 95%-CI 1.94–50, *P* = 0.0058), mainly driven by GF. For TRM, conditioning regimen was found to be the only predictor suggesting a 10-fold higher risk of TRM using a TBI based conditioning regimen (HR 10.9, 95% CI 1.2–99.2, *P* = 0.034). Age above or below the median within the pediatric cohort was not a predictor for OS, EFS or TRM.

**Table 3:** Multivariable Cox PH analysis patients <19 years old (*n* = 73).

	Overall survival		Event-free survival	
	HR (95% CI)	*P*	HR (95% CI)	*P*
Ex vivo T-cell depletion				
No	1		1	
Yes	8.39 (0.92–76.6)	0.059	2.67 (0.44–16.4)	0.29
HLA match				
Matched	1		1	
Mismatched	3.15 (0.57–17.4)	0.19	9.84 (1.94–50)	0.0058^*^

*P < 0.05.

## Discussion

With our contemporary data from three large FA referral centers, we show that survival after allo-HCT for FA (including pediatric and adult patients) is excellent, with an OS over 80%. For patients <19 years survival rates are even > 90% and up to 100% for those receiving a T-replete transplant. For the full cohort the main predictors for inferior outcome found for OS, EFS and TRM were age ≥19 years, HLA mismatch and HCT performed before 2014. In the <19-year group ex vivo TCD was found to be a borderline predictor (*P* = 0.059, HR 8) for OS as well. GvHD rates were very low across all transplant platforms (<10%).

Due to the retrospective nature of this study, one of the limitations is that reliable data on androgen use and number of transfusions received prior to HCT, factors previously described as predictive of outcomes after HCT, is missing [11, 12]. Although the definition of MDS/AML diagnosis and relapse was based on reports from institutional licensed pathologists, this was not a centralized review, which could be a limitation of this study. Another limitation is that due to center preference in general to either perform T-replete versus ex vivo TCD transplantations, it is not possible to correct for center effect and we cannot fully exclude possible variations in clinical management across centers may have contributed to outcomes. Nevertheless, we do believe our results are novel and of interest to the field, given the inclusion of a large number of patients treated in centers with particular expertise in FA and considering that a comparison between T-replete and ex vivo TCD transplants has not been reported before.

Our outcomes are consistent with or better than previously described results. In a large registry study by the European Group for Blood and Marrow Transplantation, OS was 65% at 5 years [5]; in contrast, a more recent study from Iran, including patients ≤18 years of age, showed 5-year OS of 82% [13]. These patients received in vivo TCD with rabbit anti-thymocyte globulin (ATG) and a busulfan based conditioning, without radiation. In our cohort of patients <19 years, only 26% of patients received busulfan, which may explain the higher toxicity seen in the Iranian series. This was also discussed previously by Smetsers et al., who demonstrated a 5-year OS of 87.8% in pediatric and young adult patients who received a fludarabine based conditioning regimen [3]. The prospective multi-center study by Mehta et al., including pediatric and adult patients, demonstrated a 3-year OS of 80% for recipients of alternative-donor ex vivo TCD transplant with TBI-free conditioning [6]. Other recent reports on outcomes after allo-HCT for Fanconi anemia also indicated similar outcomes as we see in our cohort, such as the Spanish multicenter study including 34 mainly pediatric patients (OS 73%) [7] and the report from Hadassah Medical Center, including 41 mostly pediatric patients (OS 82.9%) [14]

For our full cohort, a predictor for inferior survival was age ≥19 years and although the percentage of patients with MDS/AML was higher in the group ≥19 years, inferior survival was driven by TRM and not relapse. Although in our cohort only 12% of patients received TBI, in the adult group this was 50%, which may have contributed to the higher rate of TRM as described previously [5], but other factors, such as pre-treatment associated toxicities may have played a role as well. For patients <19 years, age was not found to be a predictor for any of the outcomes, which was also shown by Rostami et al. [13]. In contrast, Latour et al. and Mehta et al. both described age below and above 10 years to be an independent predictor of OS [5, 6]. Mehta showed superior OS in patients <10 years compared to patients ≥10 years, including adult patients (OS 92.3% versus 63.2%; *P* = 0.02). The more favorable OS may be explained by the generally better immune reconstitution seen in young children compared to adults in the ex vivo TCD setting, as positive viral serostatus (CMV, Adeno, EBV) is more common with increasing age. HLA mismatch and transplant performed before 2014 were also found to be multivariable predictors for inferior survival in the full cohort, but not in the cohort of patients <19 years of age, suggesting that these covariates mainly predicted outcomes in older FA patients. In addition to high resolution HLA typing, optimization of anti-microbial prophylaxis, could have contributed to better outcomes in more recent transplants.

In the <19 group, ex vivo TCD was found to be a borderline predictor for inferior survival (8-fold higher risk of an event), compared to T-replete transplants (100% OS at 5 years; *n* = 45 versus 73% in 28 TCD patients), which is in line with Smetsers et al. [3]. Although the *P* value did not reach significance at the 0.05 threshold (*p* = 0.059), the 95% confidence interval is wide and not clearly centered around 1, suggesting a lack of power, rather than a lack of effect. We acknowledge that this study was not designed to define which platform is superior, but our findings are relevant, especially in the context of recent results presented on behalf of the EBMT Severe Aplastic Anaemia Working Party and Paediatric Disease Working Party at the 64th American Society of Hematology Meeting and Exposition in 2022 [15]. Their outcomes for >800 children who underwent transplantation for FA, including only 85 (11%) ex vivo TCD transplantations, were excellent (5-year OS, EFS and GRFS to be 83%, 78% and 70%, respectively). Although ex vivo TCD is considered standard of care in some select US centers, T-replete transplantation seems to perform at least as well, and has the advantage of potential faster immune recovery reducing the risk of post transplantation complications. In addition, T-replete transplantation is not limited to centers with access to TCD and can be performed in more centers worldwide.

The rates of GvHD in both T-replete and TCD recipients are low. Ex vivo TCD is being used in some centers to minimize the risk of GvHD, however in our analyses, there was no difference in GvHD incidence between TCD and T-replete HCT, whereas TCD was associated with inferior survival.

Although follow-up time and numbers may be limited, there was no association found between a history of severe aGvHD (grade III-IV) and extensive cGvHD, and the development of malignancies later in life. This contrasts with Rosenberg et al., reporting aGvHD and cGvHD being significant risk factors for SCC in a cohort of 117 pediatric FA patients who received allo-HCT [2]. Bonfim et al. described 12 patients with FA who developed SCC after transplant, 8 of which had preceding cGvHD [16]. Although the numbers were too small to statistically determine the significance of cGvHD as a risk factor for SCC, the cohort did demonstrate that patients with preceding cGvHD developed SCC earlier. In this cohort described by Bonfim et al., the 2-year cumulative incidence of cGvHD (35%) was much higher than what we describe in our cohort. In a large cohort of pediatric and adult patients who underwent allo-HCT for any indication, Rizzo et al. reported that 0.02% of patients developed SCC after HCT; cGvHD was found to be associated with a 5-fold increased risk of SCC (0.1%) [17]. The proportion of patients with FA included was not determined.

In comparison to the study by Mehta et al. [6], describing patients who received ex vivo TCD, we show similar outcomes in a cohort that includes >50% T-replete transplants. Our data are also consistent with pediatric registry data from EBMT from 2021, demonstrating better outcomes after allo-HCT with haplo-identical transplants with only in vivo TCD (OS at 24 months 80%; *n* = 59), compared to haplo-identical transplant with both in vivo and ex vivo TCD (OS at 24 months 60%; *n* = 33) [18]. The cumulative incidence of extensive cGvHD in that analysis for haplo-identical transplants with only in vivo versus in vivo and ex vivo TCD was 3% and 4%, respectively.

In summary, we show excellent outcomes in this relatively large tri-institutional cohort of pediatric and young adult FA patients which includes the use of conventional grafts and ex vivo TCD. We show high survival and low toxicity (including low incidence of GvHD), particularly for those <19 years of age. For patients without a suitable matched sibling donor, matched unrelated donor and cord blood are good alternative cell sources. In the absence of a fully matched (un)related donor, ex vivo graft manipulation may be considered in centers with access and experience, but for other centers, alternative strategies, such as post-transplant cyclophosphamide, as explored by Bonfim et al. [19], to decrease the risk of GvHD should be considered.

### Supplementary information

Supplementary Material

## Data Availability

Data supporting the findings of the study are provided in the manuscript and supplementary data. Deidentified individual participant data reported in the manuscript will be shared under the terms of a Data Use Agreement and may only be used for approved proposals. Requests may be made to: crdatashare@mskcc.
