# Supplementary material for: Predictors of outcomes in hematopoietic cell transplantation for Fanconi anemia
Source: Bone Marrow Transplant. 2023 Oct 17;59(1):34–40. doi: 10.1038/s41409-023-02121-1 (PMC10781622; doi:10.1038/s41409-023-02121-1)
Supplement: Supplementary file 1 — Supplementary Material [file 41409_2023_2121_MOESM1_ESM.docx]

**Supplementary Information**

**Table S1**. Patient and HCT characteristics for patients < 19 years of age

| Total n=73 | n (%) |  | n (%) |
| --- | --- | --- | --- |
| **Center** |  | **Conditioning regimen** |  |
| Leiden University Medical Center | 12 (16.4) | Cy/Flu | 50 (68.5) |
| Memorial Sloan Kettering Cancer Center | 26 (35.6) | Bu^1^/Cy/Flu | 19 (26) |
| University Medical Center Utrecht/Princess Maxima Center for Pediatric Oncology | 35 (47.9) | TBI/Cy/Flu | 3 (4.1) |
| **Age at HCT** |  | Cy/Thiotepa | 1 (1.4) |
| Median (years) | 8 | **Serotherapy** |  |
| Range (years) | 1.7-18.9 | ATG | 68 (93.2) |
| **Gender** |  | Alemtuzumab | 1 (1.4) |
| Female | 26 (35.6) | None | 4 (5.5) |
| Male | 47 (64.4) | **HLA matching** |  |
| **Disease status at time of HSCT** |  | Matched | 49 (67.1) |
| Bone marrow failure +/- cytogenetic changes | 61 (83.6) | Mismatched | 24 (32.9) |
| MDS/AML | 12 (16.4) | **Donor** |  |
| **Follow-up** |  | MUD | 29 (39.7) |
| Median (years) | 3.6 | MRD | 20 (27.4) |
| Range (years) | 0.9-14.3 | MMUD | 15 (20.5) |
|  |  | MMRD | 9 (12.3) |
|  |  | **Stem cell source** |  |
|  |  | Bone marrow | 39 (53.4) |
|  |  | Peripheral blood | 23 (31.5) |
|  |  | Cord blood | 11 (15.1) |
|  |  | **Graft manipulation** |  |
|  |  | Unmanipulated/conventional | 45 (61.6) |
|  |  | *Ex-vivo* T cell depletion^2^ | 28 (38.4) |

^1^ BU target was 18-20 mg*h/L at MSKCC and 30 mg*h/L at LUMC and UMC/PMC. In the European centers Bu was only used for MDS/AML patients.

^2^T-cell depletion devices used were Isolex (n=5) and CliniMACS (n=23).

**Table S2**. Patient and HCT characteristics for patients ≥ 19 years of age

| Total n=16 | n (%) |  | n (%) |
| --- | --- | --- | --- |
| **Center** |  | **Conditioning regimen** |  |
| Memorial Sloan Kettering Cancer Center | 9 (56.2) | Cy/Flu | 1 (6.2) |
| University Medical Center Utrecht | 7 (43.8) | Bu^1^/Cy/Flu | 7 (43.8) |
| **Age at HCT** |  | TBI/Cy/Flu | 8 (50) |
| Median (years) | 30.2 | **Serotherapy** |  |
| Range (years) | 22.8-44 | ATG | 16 (100) |
| **Gender** |  | **HLA matching** |  |
| Female | 7 (43.8) | Matched | 10 (62.5) |
| Male | 9 (56.2) | Mismatched | 6 (37.5) |
| **Disease status at time of HSCT** |  | **Donor** |  |
| Bone marrow failure +/- cytogenetic changes | 9 (56.2) | MUD | 8 (50) |
| MDS/AML | 7 (43.8) | MRD | 2 (12.5) |
| **Follow-up** |  | MMUD | 5 (31.3) |
| Median (years) | 5.1 | MMRD | 1 (6.2) |
| Range (years) | 1.4-7.7 | **Stem cell source** |  |
|  |  | Bone marrow | 6 (37.5) |
|  |  | Peripheral blood | 9 (56.3) |
|  |  | Cord blood | 1 (6.2) |
|  |  | **Graft manipulation** |  |
|  |  | Unmanipulated/conventional | 7 (43.8) |
|  |  | *Ex-vivo* T cell depletion^2^ | 9 (56.2) |

^1^ BU target was 18-20 mg*h/L at MSKCC and 30 mg*h/L at LUMC and UMC/PMC. In the European centers Bu was only used for MDS/AML patients.

^2^T-cell depletion devices used were Isolex (n=3) and CliniMACS (n=6).
